# Plant volatile emission depends on the species composition of the neighboring plant community

**DOI:** 10.1186/s12870-018-1541-9

**Published:** 2019-02-06

**Authors:** Rose N. Kigathi, Wolfgang W. Weisser, Michael Reichelt, Jonathan Gershenzon, Sybille B. Unsicker

**Affiliations:** 1 Institute of Ecology, Friedrich-Schiller-University of Jena, Dornburger Str. 159, 07743 Jena, Germany; 2 Department of Biochemistry, Max Planck Institute for Chemical Ecology, Hans-Knöll Str. 8, 07745 Jena, Germany; 3 Present Address: Terrestrial Ecology Research Group, Department of Ecology and Ecosystem Management, School of Life Sciences Weihenstephan, Technische Universität München, Hans-Carl-von-Carlowitz-Platz 2, 85354 Freising, Germany; 4 Present Address: Department of Biological Sciences, Pwani University, P.O Box 195-80108, Kilifi, Kenya

**Keywords:** Biodiversity effects, Experimental grassland, *Fabaceae*, *Geraniaceae*, Herbivore-induced plant *volatiles* (HIPV), *Poaceae*

## Abstract

**Background:**

Plants grow in multi-species communities rather than monocultures. Yet most studies on the emission of volatile organic compounds (VOCs) from plants in response to insect herbivore feeding focus on one plant species. Whether the presence and identity of neighboring plants or plant community attributes, such as plant species richness and plant species composition, affect the herbivore-induced VOC emission of a focal plant is poorly understood.

**Methods:**

We established experimental plant communities in pots in the greenhouse where the focal plant species, red clover (*Trifolium pratense*), was grown in monoculture, in a two species mixture together with *Geranium pratense* or *Dactylis glomerata*, or in a mixture of all three species. We measured VOC emission of the focal plant and the entire plant community, with and without herbivory of *Spodoptera littoralis* caterpillars caged on one red clover individual within the communities.

**Results:**

Herbivory increased VOC emission from red clover, and increasing plant species richness changed emissions of red clover and also from the entire plant community. Neighbor identity strongly affected red clover emission, with highest emission rates for plants growing together with *D. glomerata*.

**Conclusion:**

The results from this study indicate that the blend of VOCs perceived by host searching insects can be affected by plant-plant interactions.

**Electronic supplementary material:**

The online version of this article (10.1186/s12870-018-1541-9) contains supplementary material, which is available to authorized users.

## Background

Plants constitutively emit volatile organic compounds (VOCs) from flowers, leaves, and roots. Emission generally increases when plants are attacked by antagonists such as insect herbivores or pathogens [26, 58]. The function of these VOCs is diverse and not fully understood. VOCs play a role in protection against abiotic stresses such as ozone, light and temperature (recently reviewed by [40]), act as attractant in biotic interactions such as pollination [6, 10, 28], or seed dispersal [50], or serve as direct or indirect defense against natural enemies [5, 6, 44] including defense priming [15, 16, 18]. There are also further functions of VOCs in intra- and interspecific plant communication [29, 30, 36].

So far, single plant individuals have been the main focus of studies investigating the role of plant VOCs in biotic interactions. Because plants most often interact with conspecific and heterospecific plants in the field, several recent reviews have suggested that studies should be expanded to more natural situations in order to understand the evolutionary roles of herbivore induced plant volatiles (HIPVs) under natural conditions [22, 47]. In particular, it seems essential to challenge plants by growing them with conspecific and/or heterospecific neighbors of different plant community complexities, such as with increasing plant diversity [31].

Recent studies have demonstrated that neighbor identity can drastically affect the defense chemistry of a focal plant species. In a study by Broz et al. [3], *Centaurea maculosa* plants growing next to conspecific neighbors contained higher levels of phenolic compounds after elicitation with methyl jasmonate than plants growing with a heterospecific grass species. Neighbor identity also significantly affected the VOC emission from the legume red clover (*Trifolium pratense*) [35]. In competition with conspecific neighbors, red clover plants emitted lower amounts of constitutive and herbivore induced VOCs as compared to those growing in inter-specific competition with the orchard grass *Dactylis glomerata* [35]. Neighbor-induced changes in plant allocation to defense compounds likely affect the resistance against biotic stresses and can thus ultimately affect the persistence of plants in a community.

Increasing plant species richness in a plant community has been shown to result in increased insect herbivory [39], increased visits of pollinators [12] and changes in more complex plant-insect interactions [13, 57]. These ecosystem-level effects of plant diversity are often the consequence of plastic responses of plants to the community in which they grow [56] and may thus also affect plant defence. While there are studies on how plant diversity affects nutrient allocation to growth and reproduction across different plant diversity levels (e.g. [21]), the effects of plant species richness on the investment of plants into defense have rarely been investigated. Mraja et al. [43] showed that iridoid glycoside defenses in ribwort plantain (*Plantago lanceolata*) were significantly affected by plant species richness in experimental grasslands. For VOC emissions, effects of plant diversity have been postulated [49], but empirical evidence is scarce (recently reviewed by [42]).

The odor released from plant communities is composed of VOCs emitted from individual plants within these communities. Host searching insect herbivores or parasitoids and predators foraging in complex plant communities are thus always confronted with a diverse mixture of plant derived VOCs. As insects have been shown to perceive even minute amounts of such VOCs [6], it seems likely that quantitative and/or qualitative changes in the VOC emission of plant communities can negatively or positively affect the host- or prey searching behavior of insects. Conversely, if a target plant under attack by an insect herbivore ‘cries for help’ with an amplified release of herbivore-induced VOCs, it’s attractiveness to a host seeking parasitoid or an insect predator may be diminished due to neighbor-inflicted masking of the plants’ VOC signals. These complexities of plant community VOC emissions and the resulting responses of searching insects are only starting to be addressed. An understanding of the role of VOCs in plant-insect interactions in the field thus requires analyzing VOC emission not only at the level of a focal plant, but also at the level of the plant community.

In this study we investigated the effects of plant species composition and plant species richness on constitutive and herbivore-induced VOC emission of the focal plant species, red clover (*Trifolium pratense*), and from the entire plant community including red clover. In an experimental greenhouse study we created grassland communities with a plant species richness gradient ranging from one to three species, by combining red clover with the forb *Geranium pratense* and the grass *Dactylis glomerata*. To analyze the effect of herbivory on VOC emission, we compared red clover fed upon by larvae of the generalist lepidopteran *Spodoptera littoralis versus* untreated control plants. Although this species is not recorded as an herbivore on red clover so far, we chose it, as it is a broad generalist that reliably consumes significant quantities of leaf material. Previous studies have successfully employed *S. littoralis* for unravelling plant responses to herbivory [33, 37] including our own studies [17, 34].

We addressed the following specific questions:

1. Is the VOC emission of red clover and of the entire plant community influenced by the composition and diversity of the surrounding plant community?
2. Are there effects of plant identity, i.e. does it matter for VOC emission if red clover grows together with *D. glomerata* or *G. pratense*?
3. Are VOC emissions from a single herbivore induced plant detectable at the community level?

## Results

### *Trifolium pratense* biomass and herbivory

The biomass of the focal *T. pratense* plants was affected by plant species richness, species composition and the identity of the neighbouring plants (Additional file 1: Figure S1A and Table S1). *T. pratense* plants growing with *D. glomerata* (TD and TDG mixtures*)* had lower biomass compared to plants in mixtures without the species, while plants growing with *G. pratense* were not different from those growing in *T. pratense* monoculture (Additional file 1: Table S1, Model 3). Additional file 1: Figure S1 illustrates that *T. pratense* individuals growing in plant species mixtures had a lower biomass than individuals growing in monocultures. The model with the lowest AIC (best fit) for *T. pratense* biomass was the one with species composition (Model 2, Additional file 1: Table S1A).

For entire plant communities, species richness, species composition and the identity of the neighbouring plants affected total biomass (Additional file 1: Figure S1B and Table S1B). Biomass was highest in the most diverse community and communities with *D. glomerata* were found to have higher biomass than other communities. The model with the lowest AIC for community biomass was the one with neighbor identity (Model 3, Additional file 1: Table S1B).

The percent leaf area loss due to *Spodoptera littoralis* caterpillar feeding was on average 18.4 ± 2.0% (calculated across treatments). Herbivory tended to increase with species richness, but this was not significant (Additional file 1: Figure S2 and Table S2 Model 1). Plant species composition and neighbor identity (presence of *D. glomerata* or *G. pratense*) did not affect caterpillar herbivory (Additional file 1: Table S2, Models 2 and 3).

### VOC emission from *Trifolium pratense*

*T. pratense* emitted very low amounts of VOCs when there was no herbivory. Upon caterpillar damage, total VOC emission from *T. pratense* was found to be three-fold higher than the emission of control plants (emissions: Table 1, statistical results on the effect of herbivory: Additional file 1: Table S3). *S. littoralis* feeding resulted in an increased emission of five compounds, 1-octen-3-ol, the monoterpenes (*E*)-β-ocimene and (*Z*)-β-ocimene), the sesquiterpene (*E*)-β-caryophyllene and 4,8-dimethyl-1,3,7-nonatriene (DMNT) (Tables 1 and 2, statistical results of the herbivory effect: Additional file 1: Table S3).

**Table 1:** Constitutive and herbivore-induced VOCs released from single individuals of the focal plant species *Trifolium pratense* growing in pot communities of differing species compositions. *T. pratense* plants were either grown in monoculture (1 sp), in two species mixtures with *Geranium pratense* (TG) or *Dactylis glomerata* (TD), or in three species mixtures with *D. glomerata* and *G. pratense* (3 spp). *T. pratense* individuals in half of the experimental pot communities were infested with *Spodoptera littoralis* caterpillars (herbivory treatment). Values represent mean VOC emissions (ng g^− 1^ h^− 1^) ± SEM from single *T. pratense* individuals

Compounds	Control					Herbivory				
	1 sp.	2 spp.	3 spp.	TG	TD	1 sp.	2 spp.	3 spp.	TG	TD
Monoterpenes*^d^										
(*E*)-β-ocimene*	0.5 ± 0.5	0.9 ± 0.6	1.7 ± 1.0	–	1.6 ± 1.1	26.7 ± 5.4	54.1 ± 5.4	59.8 ± 19.6	41.5 ± 11.0	69.2 ± 11.1
(*Z*)-β-ocimene*	–	–	–	–	–	2.8 ± 1.3	10.5 ± 2.3	9.4 ± 3.9	5.7 ± 1.9	16.3 ± 2.7
Sesquiterepenes*^d^										
(*E*)-β-caryophyllene*	–	–	–	–	–	2.3 ± 1.5	5.3 ± 2.2	6.5 ± 4.2	1.6 ± 1.1	9.7 ± 4.1
Homoterpene*										
DMNT*	–	–	–	–	–	14.3 ± 6.0	13.1 ± 5.0	15.1 ± 8.0	4.6 ± 2.2	23.2 ± 9.0
GLVs^d^										
(*Z*)-3-hexenyl acetate	4.2 ± 2.8	4.5 ± 2.4	5.7 ± 5.7	–	8.2 ± 3.7	5.9 ± 5.9	44.8 ± 19.7	21.0 ± 10.1	16.3 ± 14.8	78.9 ± 35.6
*Others*										
1-Octene-3-ol*	–	–	–	–	–	–	3.2 ± 1.7	2.4 ± 2.4	2.4 ± 2.4	4.1 ± 2.8
Benzyl alcohol	1.2 ± 1.2	7.4 ± 3.1	4.2 ± 1.9	8.7 ± 6.0	6.4 ± 3.3	2.1 ± 1.3	1.8 ± 1.1	3.6 ± 2.3	1.4 ± 0.9	2.2 ± 2.2
Unknown	2.3 ± 1.5	4.1 ± 2.1	4.8 ± 3.1	6.1 ± 3.8	2.4 ± 2.4	3.9 ± 2.6	3.6 ± 1.8	5.2 ± 3.2	3.6 ± 2.4	3.6 ± 3.0
Nonanal	5.5 ± 2.0	13.2 ± 6.1	11.4 ± 4.6	11.7 ± 7.9	14.4 ± 9.6	13.3 ± 6.4	13.2 ± 4.6	15.6 ± 7.1	11.1 ± 4.7	15.7 ± 8.9
Total*^d^	13.7 ± 4.2	30.1 ± 7.9	27.8 ± 2.6	26.5 ± 13.3	33.2 ± 10.4	71.3 ± 15.2	149.5 ± 30.3	138.4 ± 41.8	88.4 ± 17.5	223.0 ± 46.2

All data was analyzed using analysis of variance (ANOVA) following transformation of data to meet statistical assumptions. An asterisk (*) designates compounds which increased in their emission with *S littoralis* herbivory (*p* < 005) and the superscript letter (^d^) designates compounds that increased in the presence of the grass species *D. glomerata, n* = 5

**Table 2:**
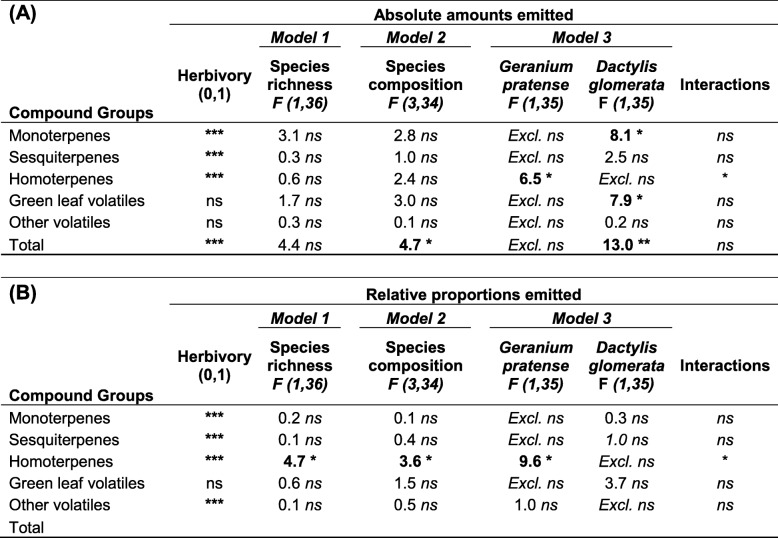
Statistical results for the analysis of VOC emissions from individuals of the focal plant species *Trifolium pratense* growing in experimental plant communities (Fig. 1). The table shows effects of species richness (1 to 3 plant species), species composition (four levels, *Trifolium pratense* monoculture (1sp), two species mixture of *T. pratense* and *Geranium pratense*, two species mixture of *T. pratense* and *Dactylis glomerata* and the three species mixture containing *T. pratense*, *G. pratense* and *D. glomerata*) and species identity (with the effect of *D. glomerata* or *G. pratense* presence shown in separate columns) on the VOC emission from single individuals of *T. pratense.* Statistical analyses were performed with absolute amounts (ng g^− 1^ h^− 1^) and relative amounts (% of the total VOC emission) of VOCs emitted. The first column in both panels (A and B) denotes the statistical results for the effect of caterpillar herbivory. As the effect of herbivory was the same in these models (where it was always fitted first), the statistical result is only presented once. Interactions between species richness, species composition and herbivory (*Spodoptera littoralis* caterpillar herbivory) are reported in the column “interactions”. Species richness, species composition and species identity (presence or absence of a species) were tested in separate analysis of variance models following transformation of data to meet assumptions of normality and homogeneity of variances (see main text for details). Statistically significant results are depicted in bold with asterisks indicating level of significance (***P < 0.001, **P < 0.01 and *P ≤ 0.05) and degrees of freedom are provided at the top of the table as F (df1, df2), n = 5. See Additional file 2: Table S4 for full models

**Fig. 1:**
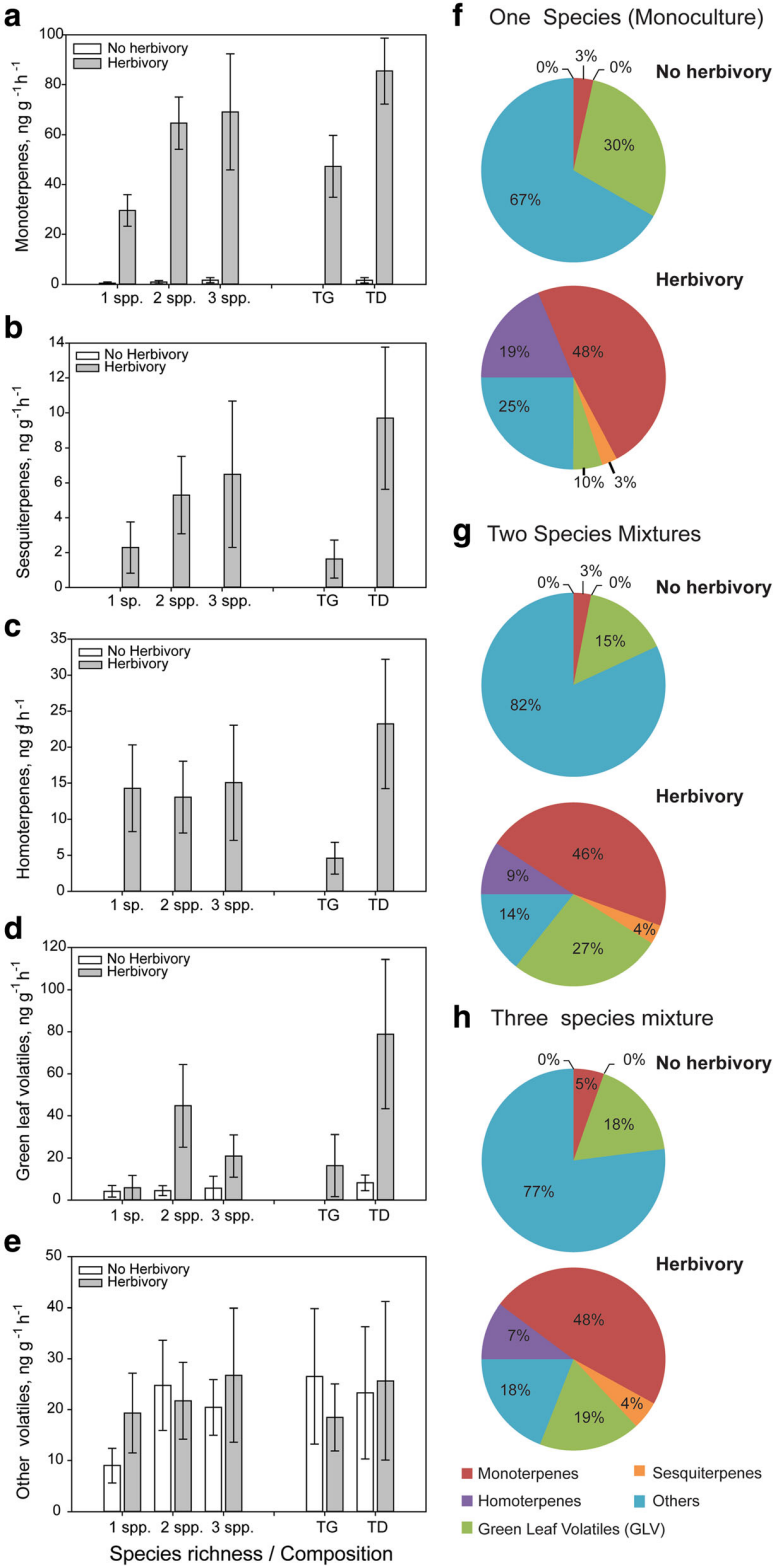
Constitutive and herbivore-induced emission of major groups of volatile organic compounds (VOCs) by single individuals of the focal plant species *Trifolium pratense* growing in pot communities of differing species compositions. *T. pratense* plants were either grown in monoculture (1 sp), in two species mixtures with *Geranium pratense* (TG) or *Dactylis glomerata* (TD), or in three species mixtures with *D. glomerata and G. pratense* (3 spp). The focal *T. pratense* individuals in the experimental communities were either exposed to feeding by three *S. littoralis* caterpillars (Herbivory, grey bars) or had no caterpillars feeding on them (Control, white bars). Values in panels (**a**, **b**, **c**, **d** and **e**) represent absolute amounts of VOCs from single *T. pratense* individuals in ng g^-1^ h^-1^ and values in the pie charts in panels (**f**, **g** and **h**) represent relative amounts of the major groups of VOCs with respect to the full *T. pratense* odour blend. Homoterpenes were only emitted by plants in the Herbivory treatment. Bars represent means ± SEM, n = 6. The results of the statistical analyses are depicted in Table 2

**Fig. 2:**
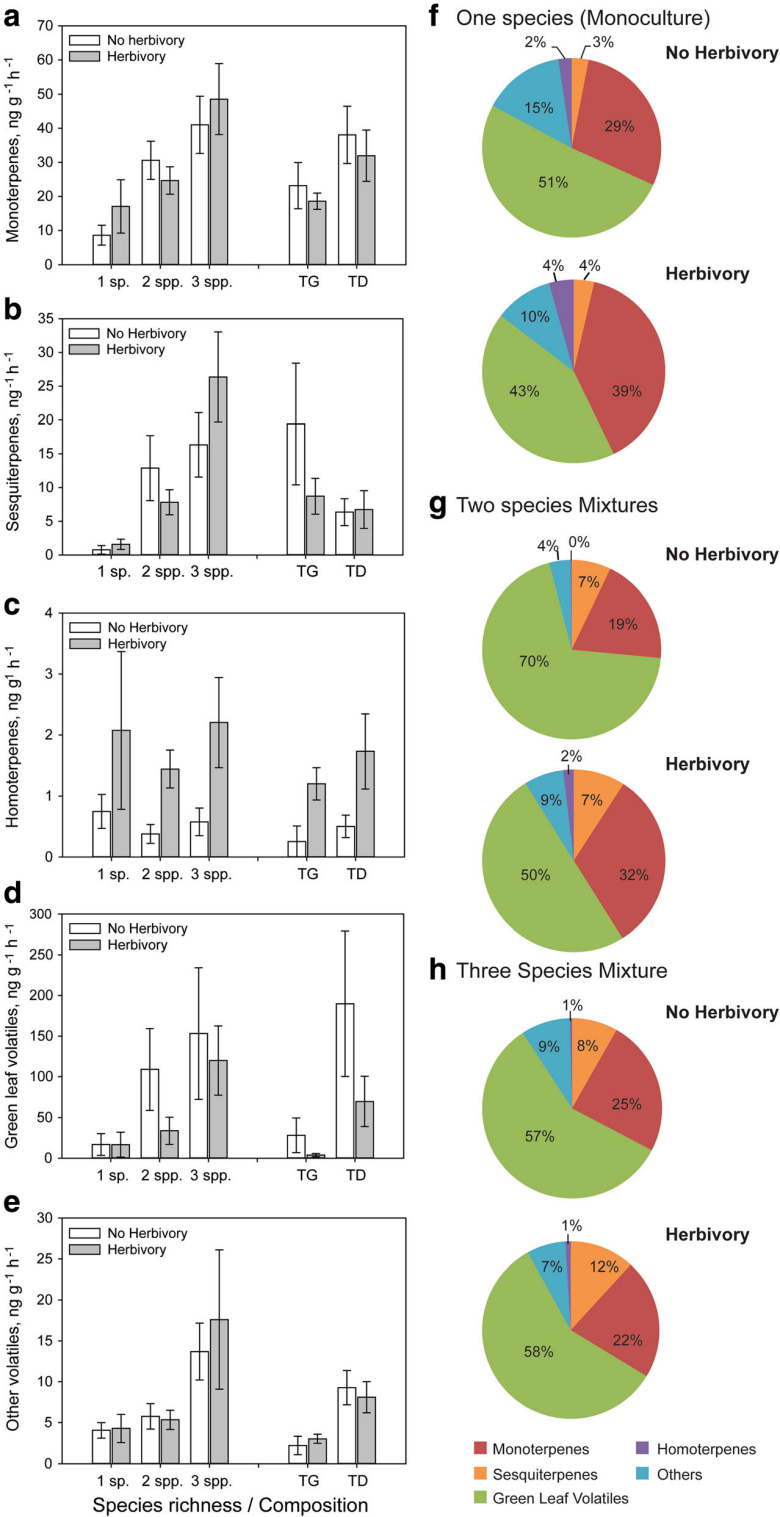
Constitutive and herbivore-induced emission of major groups of volatile organic compounds (VOCs) from experimental plant communities grown in pots in the greenhouse. *T. pratense* plants were either grown in monoculture (1 sp), in two species mixtures with *Geranium pratense* (TG) or *Dactylis glomerata* (TD), or in three species mixtures with *D. glomerata and G. pratense* (3 spp). Single individuals of the focal plant species *T. pratense* in the communities were either exposed to feeding by three *S. littoralis* caterpillars (Herbivory, grey bars) or had no caterpillars feeding on them (Control, white bars). Values in panels (**a**, **b**, **c**, **d** and **e**) represent absolute amounts of VOC emission from experimental plant communities in ng g^− 1^ h^− 1^ and values in the pie charts in panels (**f**, **g** and **h**) represent relative amounts of the major groups of VOCs with respect to the full *T. pratense* odour blend. Bars represent means ± SEM n = 6. The results of the statistical analyses are depicted in Table 4

Plant species richness did not affect VOC emission from the focal plant species *T. pratense,* (Fig. 1a-e, Tables 1 and 2A, Model 1, for full results see Additional file 2: Table S4A). Species composition had no effect on the emission of major groups of VOCs, when considered separately, but significantly affected the total amount of VOC emission (Fig. 1a-e, Tables 1 and 2A, Model 2, Additional file 2: Table S4). Neighbor identity did significantly affect VOC emission of various groups and Model 3 had the lowest AIC for all major groups of VOCs (Table 2 Additional file 2: Table S4A). The strong effect of the identity of the neighboring plants on *T. pratense* VOC emission was mainly due to the presence of *D. glomerata*. *T. pratense* plants growing in communities containing the grass species *D. glomerata* emitted higher amounts of monoterpenes and green leaf volatiles, and as a consequence of this, the overall VOC emissions from *T. pratense* in these communities was higher (Fig. 1a, d, Tables 1 and 2A, Model 3, Additional file 2: Table S4A). *T. pratense* growing together with *G. pratense* showed a significantly lower emission of the homoterpene DMNT (Fig. 2c, Tables 1 and 2A, Model 3, Additional file 2: Table S4A).

When the relative amounts (proportions) of major groups of VOCs in the full blend of *T. pratense* were considered, there were significant changes in the relative amounts of the different compound groups (Fig. 1f-h, Table 2B, for full results see Additional file 2: Table S4B). In the herbivory treatment, monoterpenes increased to 48% of the full VOC blend compared to only 3% in non-infested control plants (Fig. 1f-h, Table 2B).

Species richness, species composition and species identity did not affect the relative amounts of monoterpenes, sesquiterpenes, green leaf volatiles and other VOC released from *T. pratense* (Table 2B, Additional file 2: Table S4B). Only the relative amount of the homoterpene DMNT was affected by species richness, composition and identity (Table 2B) as *T. pratense* plants growing in two- and three-species mixtures emitted lower relative amounts of DMNT than plants growing in monocultures (Fig. 1f-h, Table 2B, Model 1). Similarly, *T. pratense* plants growing together with *G. pratense* emitted significantly lower relative amounts of homoterpenes (DMNT) as compared to other mixtures (Table 2B, Model 3, Additional file 2: Table S4B). There was a significant interaction between herbivory and the presence of *G. pratense* for homoterpene (DMNT) emission (Table 2B, Model 3, Additional file 2: Table S4B).

### *Community* VOC *emission*

On average twelve different VOCs were emitted from the entire plant communities and this did not change with caterpillar herbivory (glm with Poisson errors, Table 4A, Additional file 3: Table S5A). However, the number of VOCs produced significantly increased with plant species richness, from six compounds in *T. pratense* monocultures to 17 compounds in three-plant-species mixtures (Additional file 3: Table S5A). Similarly, both plant species composition and the identity of the neighboring plant species had a significant effect on the number of VOCs emitted (*T. pratense* monocultures released significantly fewer VOCs than the two-species mixtures and the three-species-mixture (Additional file 3: Table S5A).

The total amount of VOCs (ng h^− 1^ h^− 1^) emitted from communities was significantly affected by plant species richness, community composition and the identity of the neighboring plant species (Table 3, statistical results: Table 4A, Models 1–3, for full statistical results see Additional file 3: Table S5A). The total amount of VOCs increased significantly with plant species richness (Tables 3 and 4, Model 1, Additional file 3: Table S5A). For plant species composition (Model 2), the two species mixture of *T. pratense with D. glomerata* had higher VOC emissions compared to the two species mixture with *G. pratense* and the three-species mixture (Tables 3 and 4A, Additional file 3: Table S5A). The presence of *D. glomerata* in the two species mixture resulted in an increase in total VOC emission while the presence of *G. pratense* had no effect (Tables 3 and 4A, Model 3, Additional file 3: Table S5A). There were no significant interactions between herbivory and species richness, species composition or species identity.

**Table 3:** Constitutive and herbivore-induced VOCs released from entire experimental plant communities grown in pots in the greenhouse. 1 sp = *Trifolium pratense* monoculture, TG = two species mixture of *T. pratense* and *Geranium pratense*, TD = two species mixture of *T. pratense* and *Dactylis glomerata* (TD), 3 spp (TGD) = three species mixture with *T. pratense*, *D. glomerata* and *G. pratense*. In half of the experimental plant communities, one single *T. pratense* individual was infested with *Spodoptera littoralis* caterpillars (Herbviory). Values represent mean VOC emissions (ng g^−1^ h^−1^) ± SEM from the experimental plant communities

Compounds	Control					Herbivory				
	1 sp. (T)	2 spp.	3 spp. (TDG)	TG	TD	T (1 sp.)	2 spp.	3 spp. (TDG)	TG	TD
Monoterpenes										
(*Z*)-β-ocimene	1.5 ± 0.6	3.9 ± 1.3	4.4 ± 1.5	6.5 ± 2.2	1.3 ± 0.2	2.2 ± 1.0	3.4 ± 0.6	6.1 ± 1.4	3.9 ± 0.9	2.7 ± 0.8
(E)-β-ocimene	6.8 ± 2.3	18.8 ± 4.8	23.6 ± 4.7	7.6 ± 2.2	30.1 ± 6.9	13.4 ± 6.0	17.3 ± 3.4	33.4 ± 8.2	11.0 ± 1.4	24.9 ± 5.9
Tricyclene	–	2.2 ± 0.9	1.6 ± 0.7	4.4 ± 1.3	–	–	1.1 ± 0.5	2.3 ± 0.5	2 ± 0.7	–
α-Pinene	0.2 ± 0.2	1.2 ± 0.4	1.4 ± 0.3	2.0 ± 0.6	0.3 ± 0.2	0.1 ± 0.1	0.7 ± 0.2	1.3 ± 0.2	1.2 ± 0.2	0.1 ± 0.1
Camphene	–	0.2 ± 0.1	0.3 ± 0.2	0.5 ± 0.2	–	–	0.1 ± 0.1	0.3 ± 0.1	0.1 ± 0.1	–
β-pinene	–	0.3 ± 0.1	0.5 ± 0.2	0.7 ± 0.2	–	–	0.1 ± 0.1	0.5 ± 0.2	0.2 ± 0.1	–
Myrcene	–	1.4 ± 0.5	3.5 ± 1.4	1.0 ± 0.3	1.9 ± 0.8	–	0.8 ± 0.4	1.9 ± 0.6	0.2 ± 0.2	1.6 ± 0.8
Limonene	0.1 ± 0.1	2.0 ± 0.6	4.3 ± 1.4	0.4 ± 0.2	3.7 ± 0.8	0.3 ± 0.3	0.7 ± 0.3	2.2 ± 0.5	0.1 ± 0.1	1.3 ± 0.6
Linalool	–	0.5 ± 0.3	1.4 ± 0.4	0.2 ± 0.2	0.9 ± 0.6	1.1 ± 1.0	0.6 ± 0.4	0.7 ± 0.4	–	1.4 ± 0.7
Sesquitepene										
(E)-β-Caryophyllene*	0.1 ± 0.1	3.5 ± 1.8	3.8 ± 1.0	5.9 ± 3.3	1.1 ± 0.4	1.6 ± 0.8	3.0 ± 0.6	9.7 ± 2.1	3.5 ± 0.9	2.5 ± 0.9
(E)-β-Farnesene	–	2.2 ± 1.4	1.8 ± 1.2	4.3 ± 2.6	–	–	0.4 ± 0.3	3.7 ± 2.0	0.8 ± 0.5	–
cucurmene	–	1.7 ± 1.4	0.5 ± 0.2	3.4 ± 2.7	–	–	1.3 ± 1.0	1.1 ± 0.5	2.4 ± 1.7	–
Germacrene D	–	2.8 ± 1.6	4.2 ± 2.9	5.7 ± 2.8	–	–	1.2 ± 0.4	5.9 ± 1.4	2.1 ± 0.4	0.1 ± 0.1
(E,E)-α-Farnesene	0.7 ± 7	2.7 ± 1.2	6.1 ± 2.2	0.1 ± 0.1	5.2 ± 1.8	–	1.9 ± 1.1	6.1 ± 2.4	–	4.2 ± 1.9
Homoterpenes										
DMNT*	0.8 ± 0.3	0.4 ± 0.2	0.6 ± 0.2	0.3 ± 0.3	0.5 ± 0.2	2.1 ± 1.3	1.5 ± 0.3	2.2 ± 0.7	1.2 ± 0.3	1.7 ± 0.6
GLVs										
(Z)-3-Hexenyl acetate	17 ± 13	109 ± 50	153 ± 81	28 ± 21	190 ± 90	17 ± 15	34 ± 17	120 ± 43	4 ± 2	70 ± 31
Other volatiles										
1-Octen-3-ol	–	1 ± 0.3	2.6 ± 0.7	–	2.0 ± 0.3	–	0.9 ± 0.4	2.2 ± 0.6	–	1.9 ± 0.4
Octly-acetate	–	0.2 ± 0.1	0.5 ± 0.2	–	0.4 ± 0.2	0.5 ± 0.4	0.7 ± 0.2	0.3 ± 0.2	0.7 ± 0.4	0.6 ± 0.2
Nonanal	1.8 ± 0.7	1.4 ± 0.4	1.6 ± 0.7	0.9 ± 0.5	1.9 ± 0.6	1.9 ± 1.1	1.3 ± 0.3	1.6 ± 0.6	1.6 ± 0.5	1.0 ± 0.3
Benzyl alcohol	1.9 ± 1.0	2.5 ± 1.1	8.1 ± 2.8	1.1 ± 0.7	4.0 ± 1.9	1.0 ± 0.7	1.8 ± 0.8	12.2 ± 7.7	0.4 ± 0.3	3.5 ± 1.5
Unknown	0.5 ± 0.2	0.3 ± 0.1	0.7 ± 0.2	0.3 ± 0.1	0.3 ± 0.1	0.9 ± 0.5	0.5 ± 0.2	0.6 ± 0.2	0.4 ± 0.2	0.6 ± 0.3
MESA	–	0.4 ± 0.3	0.3 ± 0.3	–	0.8 ± 0.6	–	0.3 ± 0.2	0.8 ± 0.5	–	0.6 ± 0.3
Total	31 ± 14	159 ± 55	225 ± 90	73 ± 35	244 ± 96	42 ± 20	73 ± 22	215 ± 50	35 ± 6	118 ± 40

All data was analyzed using analysis of variance (ANOVA) following transformation of data to meet assumptions of normality and homogeneity of variances. An asterisk (*) designates compounds which increased in their emission from the plant community when one individual of the focal plant *T pratense* within the community was infested with *S littoralis* caterpillars (*p* < 0.05), *n* = 5

**Table 4:**
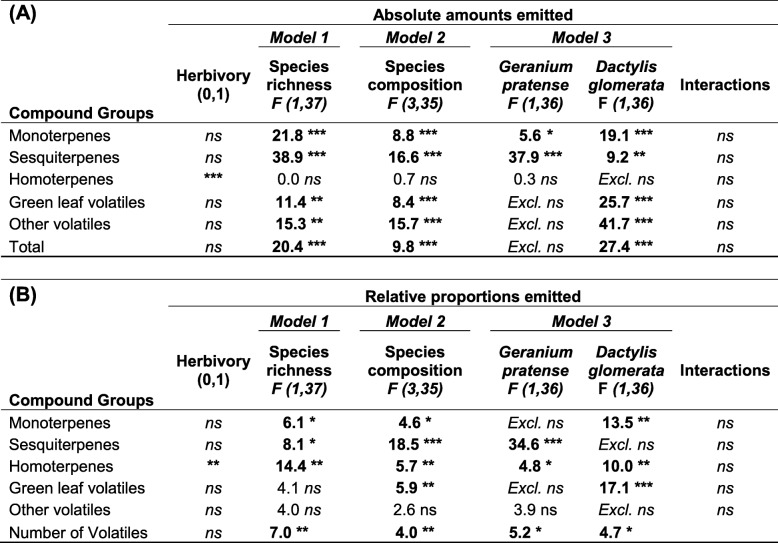
Statistical results for the analysis of VOC compound group emissions from experimental plant communities grown in pots in the greenhouse (Fig. 2). The table shows effects of species richness (1 to 3 plant species), species composition (four levels, *Trifolium pratense* monoculture (1sp), two species mixture of *T. pratense* and *Geranium pratense*, two species mixture of *T. pratense* and *Dactylis glomerata* and the three species mixture containing *T. pratense*, *G. pratense* and *D. glomerata*) and species identity (with the effect of *D. glomerata* or *G. pratense* presence shown in separate columns) on the VOC emission from experimental plant communities. Statistical analyses were performed with absolute amounts (ng g^− 1^ h^− 1^) and relative amounts (% of the total VOC emission) of VOCs emitted. The first column in both panels (A and B) denotes the statistical results for the effect of caterpillar herbivory. As the effect of herbivory was the same in these models (where it was always fitted first), the statistical result is only presented once. Interactions between species richness, species composition and herbivory (*Spodoptera littoralis* caterpillar herbivory in one individual of the focal plant species *T. pratense* in each community) are reported in the column “interactions”. Species richness, species composition and species identity (presence or absence of a species) were tested in separate analysis of variance models following transformation of data to meet assumption of normality (see text for details). Statistically significant results are depicted in bold with asterisks indicating level of significance (***P < 0.001, **P < 0.01 and *P ≤ 0.05) and degrees of freedom are provided at the top of the table as F (df1, df2), n = 5. See Additional file 3: Table S5 for full models

With increasing plant species richness in the plant communities, the emission of all major groups of VOCs except the homoterpenes (DMNT) increased significantly (Table 4A, Fig. 2a-e). Similarly, species composition and neighbor identity altered the emission of all major groups of VOCs except homoterpenes (Table 4A, Fig. 2a-e, Model 2 and 3). The emission of monoterpenes, green leaf volatiles and “other” VOCs was affected by species composition and was highest in the three species mixture and in the two species mixture of *T. pratense* and *D. glomerata* (TD) (Fig. 2a, d, e, and Additional file 3: Table S5A), while sesquiterpene emission was highest in the three species mixtures and the two species mixture with *G. pratense* (Fig. 2b, Table 4A). Only the homoterpene emission (DMNT) increased upon caterpillar herbivory and this was true in all different species combinations (Fig. 2c, Table 4A, Additional file 3: Table S5A).

The relative amounts of monoterpenes and homoterpenes in the total VOC blend decreased with increasing plant species richness while the relative amount of sesquiterpenes increased with plant species richness (Fig. 2f-h, Table 4B, Model 1, Additional file 3: Table S5B). The relative amounts of VOCs emitted from the mixtures were also strongly dependent on the composition of the plant mixtures and the identity of the plants (Table 4B, Models 2 and 3, Additional file 3: Table S5B). The presence of *G. pratense* increased the relative amount of sesquiterpenes, and decreased the relative amounts of homoterpenes (DMNT) (Table 4B, Model 3). The presence of *D. glomerata* increased the relative amount of monoterpenes and green leaf volatiles in mixtures (Table 4B, Model 3)*.*

## Discussion

In this study we investigated how the identity of neighboring plant species and plant species richness in the community affect the constitutive and herbivore-induced volatile emission from both the focal plant *Trifolium pratense* (red clover) and entire plant communities containing red clover plants. In an experimental greenhouse study we found that the VOC emission from *T. pratense* was higher in plant species mixtures and was strongly influenced by plant species composition of the surrounding plant community. When growing in communities with the orchard grass *Dactylis glomerata, T. pratense* plants emitted significantly higher amounts of constitutive and herbivore induced volatiles as compared to plants growing together with *Geranium pratense*, indicating that neighbor species identity strongly impacts the VOC phenotype of *T. pratense*.

### Herbivore induced VOC emission from red clover and the experimental plant communities

As numerous other studies before, we found a strong effect of insect herbivory of the VOC emission of the plant attacked. *S. littoralis* feeding resulted in an increased emission of five compounds, 1-octen-3-ol, (*E*)-β-ocimene, (*Z*)-β-ocimene, (*E*)-β-caryophyllene and 4,8-dimethyl-1,3,7-nonatriene (DMNT) of *T. pratense* plants. These VOCs are emitted by a wide range of plant species and some of them have been shown to attract herbivore natural enemies [5, 44]. 1-octen-3-ol was so far mostly found to be released by pathogenic or endophytic fungi (e.g. [14, 55, 61]) however, recent studies have shown that this compound is also emitted from a number of legume species such as *Vigna unguiculata* (cowpea, [2]), *Medicago truncatula* [38], *Trifolium pratense* [34] and *Lotus japonicus* [46]. The slightly higher levels of 1-octen-3-ol measured in the headspace of herbivore-infested *T. pratense* plants may be due to herbivore-inflicted damage of fungal tissue present inside/on the foliage of red clover plants. Monoterpenes showed the highest relative increase in emission after caterpillar herbivory and emission levels increased from an average of 3 to 47% across all diversity levels. (*E*)-β-Ocimene was the dominant monoterpene emitted from red clover, as reported previously [34, 35]. In contrast, in our study, methyl salicylate (MeSA) was not detected in the headspace of *T. pratense* plants. This could be either due to genotype-specific effects on VOC emission, or simply due to shorter VOC collection periods (too short to trap detectable amounts of MeSA) in our study as compared to earlier VOC collections by Kigathi et al. [34, 35]. However, Kigathi et al. [35] also showed that plants growing in intra- or inter-specific competition emitted lower amounts of MeSA than plants growing alone, which could also explain why we did not detect this compound here.

Of the five compounds that increased in VOC emission with herbivory in *T. pratense* plants, two compounds, DMNT and (*E*)-β-caryophyllene, also significantly increased in the headspace of the plant communities containing only one herbivore infested *T. pratense* plant. This was measurable despite the fact that the two compounds were emitted in relatively low amounts as compared to other VOCs emitted from the communities such as (*Z*)-3-hexenylacetate, (*E*)-β-ocimene and the aromatic compound benzyl alcohol. As specifically DMNT is indicative of insect herbivore damage (e.g. [4, 17]) this compound may function as a valuable information telling host seeking predatory insects in complex plant communities where their prey is located. Future experiments in natural grassland communities have to validate whether this assumption holds true.

As a caveat, plant species richness in our study only ranged from 1 to 3 species and there we focused on *T. pratense*, i.e. we did not investigate monocultures of *D. glomerata* or *G. pratense*. Consequently, plant species richness effects were confounded with effects of plant species composition and the presence of particular neighboring species of *T. pratense*. We partly accounted for this by using three different statistical models, each testing for one particular effect (species identity, species composition and species richness), and compared which one explained most of the variance in the VOC data. However, despite the shortcomings of the design for detecting plant diversity effects independent of plant species composition or identity, the effects of plant species richness we found are significant, as they emphasize that increased complexity of the plant community and hence an increasing number of plant-plant interactions will affect plant VOC emission and the VOC blend that a searching insect herbivore is exposed to.

### Plant species richness-, species composition- and neighbor identity effects on *Trifolium pratense* VOC emission

Increasing plant species richness in communities is known to not only introduces plant interspecific competition, but also changes a number of other parameters potentially affecting plant growth such as the interaction of plants with soil microbes or aboveground and belowground animals, or the availability of nutrients due to changes in C, N and P cycling [60]. Plants have been shown to respond to increasing plant diversity by adjusting morphological traits such as size, C:N ration or module number, as well as physiological traits such as foliar 13C values [8]. We found that VOC emissions of *T. pratense* increased with increasing plant species richness in the experimental plant communities; however, the relative amounts of individual VOCs in the blend did not change. Monoterpenes, for example, were emitted in relative amounts of 48% across all diversity levels.

The effect of plant species composition on *T. pratense* VOC emission was stronger than the effect of plant species richness, and the most striking changes in VOC emission were observed in the communities containing the orchard grass *Dactylis glomerata*. In these communities, significantly more VOCs were emitted from *T. pratense*. Thus, *T. pratense* responded to the increase in the competitive environment by upregulating its VOC emission. Recent evidence from the literature supports the finding that competitive interactions between plants can substantially change VOC emissions even at the constitutive level ([31, 47] and references therein). In an earlier study we already showed that competitor identity significantly changed the VOC emission from *T. pratense* [35].In that study, *T. pratense* plants growing with conspecifics also emitted a lower amount of VOCs than plants growing in inter-specific competition with the orchard grass *D. glomerata*, and this was even true when plants had contact only aboveground, i. e. where leaves were in contact, or only belowground, i. e. root contact [35]. In contrast to *D. glomerata*, the presence of *G. pratense* did not influence the VOC emission of *T. pratense* plants. In two-species mixtures with *G. pratense*, the abundance of VOCs in the single plant headspace was comparable to *T. pratense* plants growing in monocultures.

The proximate underlying mechanisms of these VOC emission changes are not yet clear. Neighbor-mediated changes in abiotic conditions such as water, light and nutrient availability may have affected *T. pratense* VOC emission as these factors have been shown before to up- or down-regulate plant VOC emission [20, 32]. For example, it is likely that in communities with *D. glomerata T. pratense* plants experience stronger competition [25]. Schmelz et al. [52] found that nitrogen deficiency increased the emission of sesquiterpenes from maize plants, while Gouinguene & Turlings [20] reported a decrease in VOC emission from maize under low nutrient conditions. There is also the possibility of VOC catabolism (breakdown of VOCs) within *T. pratense* individuals that can modify overall VOC emission (Reviewed by [45] TIPS) and should thus be integrated in future considerations on overall carbon flux in plants. Alternatively, the interactions between *T. pratense* and other organisms might be changed, with consequences for VOC emissions. Ballhorn et al. [1] showed that rhizobia influence HIPV emission of lima bean plants. As *T. pratense* in our study is also a legume associated with rhizobia, it is conceivable that changes in VOC emission in different experimental plant communities are an indirect effect of rhizobial densities in the roots mediated by competitive interactions with other plant species in the community [51]. Recent studies have also shown that VOC emission in plants can be induced via mycelial networks of mycorrhizal fungi transferring chemical cues from neighboring plants (reviewed by [27]). Further sources of variation for focal plant VOC emission are discussed in a recent review by Meiners [41].

### Species composition and plant species richness effects on plant community VOC emission

One of the striking results of our experiment is the effect of plant species richness and plant species composition on the VOC emission of the entire plant community. Here, the effect of plant species richness on community VOC emission was even stronger than that of plant species composition. First, the number of VOCs in the headspace increased with increasing plant species richness, which in itself is not very surprising given that each plant species is capable of emitting a specific blend of VOCs (including species-specific and non-species-specific VOCs). However, to our knowledge, this study is the first to demonstrate that VOC complexity in the headspace of plant communities increases with increasing plant species richness. Second, the total amount of VOCs in plant species mixtures also increased in comparison to *T. pratense* monocultures. Similar to the case of *T. pratense* VOC emissions, there were strong effects of plant species composition on the VOC blend of the entire plant community. The *T. pratense- D. glomerata* mixture (TD) emitted higher amounts of VOCs than the *T. pratense – G. pratense* (TG) mixture, and the composition of the blend in the headspace also depended on which plant species grew together with *T. pratense*. As in the case of emissions by single *T. pratense* it is not clear if the proximate mechanism for plant community VOC emission involves alterations in abiotic factors such as light, water and nutrients (reviewed by [31]).It is also possible that *T. pratense* plants as low VOC emitters growing in mixtures with *D. glomerata* adsorb excessive VOCs from the environment (e.g. released by high VOC emitters such as *D. glomerata*) and release them again [24, 45], leaving the erroneous impression that there is a higher production of VOCs in *T. pratense* growing in competition with the grass species. Future studies have to achieve further mechanistic understanding of the VOC emission patterns observed in our study. Although community headspace measurements could not exclude volatiles from the soil/roots, we were comparing detectable changes with diversity of headspace volatiles. This would mean that even if some emissions are from soils/roots and changes with diversity at detectable amounts, they still have potential ecological significance in the field as they potentially affect the behavior of both herbivorous insects and their natural enemies.

### Ecological consequences

Herbivore-induced plant volatiles (HIPVs) represent phenotypically plastic responses of plants to damage by insect herbivores that can result in changes in insect-plant interactions at the community level (reviewed by [47, 54]). HIPVs attract natural enemies of insect herbivores [44], but it is still unclear how this biological control functions in complex plant communities [9]. Parasitoids could require more time to find their host-plant complex in mixtures compared to monocultures, especially when neighboring plant species release similar VOC compounds [19]. However, the few studies that have examined this found little effect of increasing odor complexity on host searching efficiency of predators and parasitoids [11, 48, 59].

Natural enemies can use ubiquitous HIPVs in a ratio-specific way and may thus discriminate between different plant species, or between herbivore-wounded and healthy plants, based on the relative amounts of different VOCs investigated [5]. The analysis of headspace affects is complicated because insects do not necessarily react to the dominant compounds in the VOC profile. For example, it has recently been shown that a braconid parasitic wasp (*Glyptapanteles liparidis*) parasitizing generalist gypsy moth caterpillars is attracted to minor nitrogenous compounds released from black poplar trees from the site of actual caterpillar damage, rather than to major HIPVs such as DMNT, (*E*)-β-ocimene or sesquiterpenes[4].

In our study the highest changes in absolute amounts of VOCs in the focal plant were recorded for (*E*)-β-ocimene, and at the community level for DMNT and (*E*)-β-caryophyllene. The latter two compounds were emitted in relatively low amounts, but they were the most characteristic compounds indicative of herbivore damage. Additional VOC emissions from the single herbivore-infested *T. pratense* individuals in the community of six plants were sufficient to change the bouquet of the whole plant community, and may thus also be enough to attract natural enemies.

Overall, the differences in VOC emissions detected by comparing the emission of individual plants to emissions of the plant community level suggests that it is necessary to not only measure plants in isolation, but also measure community VOC emissions. DMNT was only emitted from the experimental plant communities containing a caterpillar-damaged red clover plant, and thus this compound may be a reliable indicator for the presence of insect herbivores also in complex plant communities. VOCs produced by plant neighbors (competitors) have been proposed to have a masking, repellent, enhancing or no (neutral) effect depending on insect sensitivity to the VOCs from non-host plants [23, 53]. In a study by Waschke et al. [59] parasitoids of the weevil *Mecinus pascuorum* feeding on ribwort plantain were not affected by additional non host-plant VOCs, but preferred the odor blend released from the host-host-plant system over the more complex blend. To ultimately elucidate the role of neighbor-released VOCs for parasitoid and insect natural enemy attraction to a focal plant, further sophisticated behavioral studies under lab and field conditions are needed.

The question arises why plants should change their VOC emission when they grow together with other plant species in a community. *T. pratense* plants growing in monocultures always emitted lower amounts of constitutive and herbivore induced VOCs than individuals growing in plant species mixtures. When the VOC emission of all plant individuals in the experimental plant communities was measured, *T. pratense* monocultures also exhibited the lowest overall emissions. One possible explanation is that plants growing in a highly complex and competitive plant community with a more complex and intensive odor environment need to increase their VOC emission, to be sensed by beneficial insects such as pollinators and parasitoids or predators of their natural enemies. In addition, when plants are attacked by herbivores, they become competitively weaker, making it even more pressing to call for herbivore natural enemies. Field studies testing the effectiveness of parasitoid attraction in simple and complex odor environments are needed to test this hypothesis.

## Conclusion

Our study revealed complex changes in the blend of VOCs released from plant communities depending on the number of plant species and the plant species composition within the community with and without insect herbivores. The challenge for future experiments is to reveal the underlying mechanisms leading to neighbor-related changes in focal plant VOC emission and if these changes convey a fitness advantage for the plant. Whether plant community and plant species richness inflicted changes in VOC emission affect the infochemical network involved in attraction of herbivores, herbivore enemies and pollinators in plant communities is also worth detailed investigation.

## Methods

### Plant and insect material

Three common Eurasian grassland species, the legume *Trifolium pratense* L., the forb *Geranium pratense* L. and the grass *Dactylis glomerata* L. were grown from seeds (Rieger Hofmann, Blaufelden-Raboldshausen, Germany) in the greenhouse, planted in plug trays filled with commercially available soil (TonKultur Subtrat®, Klassman Deilmann, Geeste, Germany). After 3 weeks, the seedlings were transplanted in groups of six individuals in pots with a diameter of 13 cm filled with commercially available potting soil TonKultur Subtrat®, mixed with sand at a ratio of 2:1. For 4 weeks the plants were maintained in the greenhouse (day: night temperatures, 20–22 °C:18–20 °C; 30–55% humidity, 16 h light, photosynthetically active radiation approximately 180 μmol m-2 s-1 at plant height). By the time the experiment started, the focal plant species *T. pratense* started budding. The aboveground biomass of the plants in the different experimental plant communities is shown in an additional graph (see Additional file 1). The three species we selected for the experiments also naturally occur together in Central European grasslands of different diversity levels (Stein et al. 2010; Unsicker et al. 2006).

*Spodoptera littoralis* Boisd. (Lepidoptera: Noctuidae) caterpillars were hatched from eggs (Syngenta, Basel, Switzerland) and reared on agar-based artificial medium [17] at 23–25 °C with a 16/8 h light/dark cycle until they reached the 3rd larval instar.

### Plant diversity and herbivory treatments

**Fig. 3:**
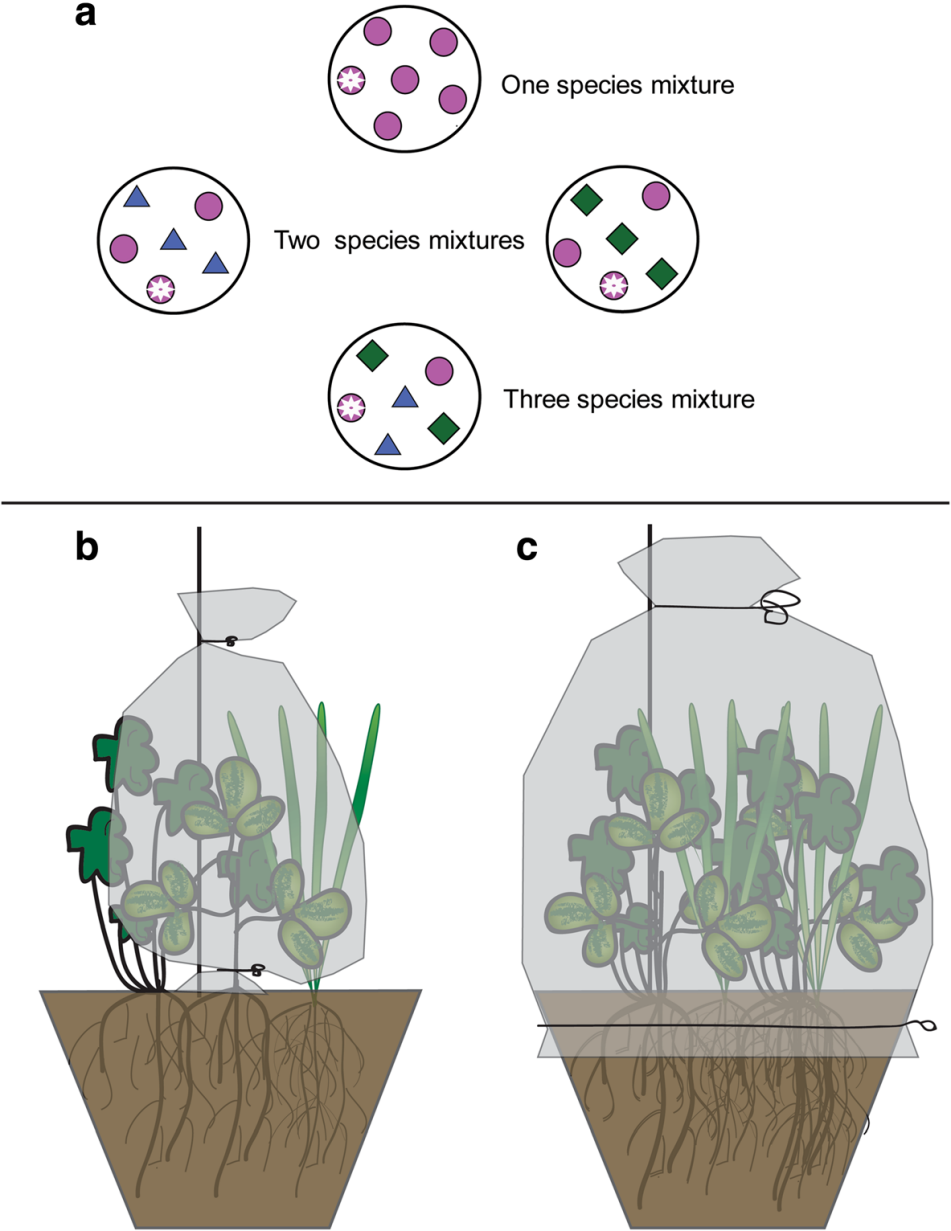
Graphical illustration of the planting scheme in the greenhouse (**a**) and the PET bag installation for VOC collections from single *Trifolium pratense* individuals (**b**) and entire experimental plant communities. (**c**) Different shapes represent different species, circles represent *T. pratense*, triangles *Geranium pratense* and squares are *Dactylis glomerata* plants Circles with an asterisk represent the individual of the focal plant species *T. pratense* that was sampled for volatile emission

The effect of plant diversity on VOC emission from the focal plant *T. pratense* and plant communities at different diversity levels was tested by varying plant species richness from one to three species. The monoculture consisted of only *T. pratense* (T), the two species mixtures had two sets: one mixture containing *T. pratense* and *D. glomerata* (TD), and the other *T. pratense and G. pratense* (TG); all three plant species were part of the three species mixture (TDG). The total number of different plant communities was therefore four. Each pot contained six plant individuals (monoculture: six of the same species, two species mixture: three of each species, three species mixture: two of each species) Fig. 3.

From each community a focal red clover plant was selected to which the herbivory treatment and VOC collections were applied. We always selected one of the outer plants in the pot to simplify bagging, herbivore application and VOC collections. Half of all pots in each diversity level were infested with herbivores (H) and the other half functioned as the non-infested control group (C). In the herbivory treatment (H), three *Spodoptera littoralis* caterpillars previously starved for 8 h were allowed to feed on the focal red clover plant for 24 h before the start of VOC collection. To restrict caterpillar feeding to the focal plant, these plants were enclosed in open top polyethylene terephthalate (PET) bags. The PET bags were very slippery and effectively prevented escape of *S. littoralis* caterpillars. For experimental consistency focal plants in the control group were also enclosed with a PET bag. In the greenhouse, the pots were grouped in six blocks, each block containing every treatment and plant composition level (2 × 4 = 8 plants in two trays). In total there were 6 × 8 = 48 pots in the experiment. The position of pots within a block was randomized. Because of the high workload involved in measuring VOCs, the herbivory treatment and subsequent VOC collection were staggered over a period of 6 days, i.e. every day one block of plants was exposed to caterpillars and VOCs were collected the following day. For VOC collections, plants of one block were transferred from the greenhouse to a climate chamber (23 °C, 16/8 h light and dark cycles, 60% relative humidity).

### VOC collection and analysis

*Single plant*: VOC emission of one *T. pratense* individual in each experimental plant community was measured using a push-pull system. Single plants were enclosed with PET cooking bags (30 cm × 60 cm, volume 28.89 ± 0.47 L, Toppits® Bratschlauch, Melitta, Minden, Germany) supported by rods of stainless steel (Fig. 3). Since the bag was tied at both ends using PET strips to form an ellipsoid, the bags had an approximate volume of 28.89 ± 0.47 L. Compressed air (from an in-house system in the greenhouse) entered the system after passing through an activated charcoal filter on the lower side of the plant at a flow rate of 4.0 ± 0.5 l/min and was pulled out at the top through an adsorbent super-Q filter (Alltech, FL, USA), using vacuum at the rate of 2 ± 0.5 l/min for 1 h. Before the initiation of VOC sampling, charcoal-filtered clean air was flushed though the collection system for 15 min to remove VOCs resulting from handling of the plants during setup of the apparatus. All volatile collections were performed between 1000 and 1300 h.

*Plant community*: After VOCs were collected from the headspace of individual *T. pratense* plants, VOCs from the headspace of the entire plant communities were collected by enclosing all plants in each pot with PET cooking bags of size 30 cm × 80 cm (approximate volume of 37.7 ± 0.47 L, Fig. 3). VOCs were collected for 1 h as described above. After VOC collection the traps were eluted with 150 μl hexane containing nonylacetate as the internal standard (concentration: 4 ng/μl; Sigma-Aldrich, Germany). As for VOC collections from the headspace of entire plant communities, PET foil was wrapped around the pots (Fig. 3), it is possible that soil-derived VOCs were also trapped.

VOCs were analyzed using an Agilent 6890 Series gas chromatograph (Agilent, Santa Clara, CA, USA), with helium as the carrier gas; the outlet of the column (DB-5MS, 30 m × 0.25 mm × 0.25 μm film, m, J & W Scientific, Folsom, CA, USA) was coupled to an Agilent 5973 N quadrupole mass detector. Parameters for electron impact sample ionization were as follows: interface temperature, 280 °C; repeller, 30 V; emission, 34.6 μA; electron energy, 70 eV; source temperature, 230 °C. The chromatographic conditions were as follows: splitless injection at 220 °C, initial oven temperature, 40 °C for 3 min, increased at 5 °C/min to 210 °C followed by an increase of 60 °C/min to 300 °C and held for 2 min. We used commercially available authentic standards (Sigma Aldrich, St. Louis, MO, USA) for the identification of all VOCs listed in Table 1 and Table 3 and in case of Germacrene D, Curcumene and (*E*,*E*)-α-Farnesene, where we did not have standard compounds, we verified the VOC identity by calculating Kovats retention indices. Additionally, mass spectra were compared with those in the NIST and Wiley mass spectra libraries. Individual compounds were quantified by comparing the peak areas in the FID traces with that of the internal standard (nonyl acetate) calculated according to the effective carbon number concept described by Scanion and Willis (1985).

### Plant harvest and herbivory measurements

After VOC collection, herbivory of the focal *T. pratense* plants was estimated visually. Thus, percent leaf area loss in each of the three leaflets of a leaf (between 20 and 30 leaves in each plant) was scored and then an average leaf area loss per leaf was calculated for each plant individual. After herbivory had been estimated, focal *T. pratense* were harvested separately from the rest of the community by cutting the plant 3 cm above the ground. The harvested plants were freeze-dried and the dry weight of each individual was then determined. The other plants in the community as well as control plant communities were also harvested, pooled by species and the dry weight was determined after plants were dried for 48 h at 70 °C. VOC measurements and biomass harvesting were performed on separate days for the different blocks.

### Statistical analysis

All analyses were carried out using the software package R (https://www.r-project.org/), version 3.4.1. All statistical assumptions such as homogeneity of the variances and normality were checked and the VOC emission data of the focal *T. pratense* plants (ng g^− 1^ h^− 1^) was log-transformed before statistical testing. The proportions of VOCs emitted were calculated as proportions of total emission and arcsine transformed before statistical analysis. The data was then analyzed using analysis of variance (aov), except for the numbers of compounds emitted from the community, which were analyzed using a generalized linear model (glm) with poisson error distribution, because the count data were not normally distributed.

In our design, plant species richness (1–3 plant species), composition (T, TD*,* TG, TGD), and the presence of a plant species additional to *T. pratense* (i.e. D or G) were confounded in our design, e.g. increasing plant species richness was only possible be including an additional plant species. This is because we only had three species in our experiment. Thus, we cannot put all terms (species richness, composition, presence of particular species) into the same model. Nevertheless, it is important to understand which of these different explanatory variables has the largest effect. After consulting statisticians, we decided for the following approach in the statistical analysis: for each dependent variable, we run different statistical models (procedures aov and glm in R, depending on the variable type, see above), one focusing on plant species richness (model 1), one on species composition (model 2) and one on neighbor species identity (model 3), i.e. the species *G. pratense* and *D. glomerata*. We run each model separately and compared their results using the AIC, to see which model explained the patterns best, i.e. had the lowest AIC. However, because we know that that the different types of independent variables are confounded, we are careful not to state that one variable (e.g. species richness) is more important than the other (e.g. composition), rather, we neutrally state if a particular variable has a significant effect and how this effect compares to the effect of the others.

Thus, the data was analyzed as follows: the effects of species richness (3 levels), species composition (four levels) and neighbor species identity (presence/absence of *D. glomerata* and *G. pratense*) on VOC emissions of focal plants, or the entire community, were tested in separate analyses of variance models with each factor as the main effect, and block as a random effect. Interaction effects between herbivory and species richness and herbivory and species identity were also tested. In our statistical analysis, we followed the procedure of model simplification [7]: the models were simplified until the minimum adequate model was achieved. The initial models were as follows:

- Model 1 (species richness):

y~Block + Herbivory + Species richness+ Herbivory * species richness

- Model 2 (species composition):

y~Block +Herbivory+ Species composition + (Species composition *Herbivory)

- Model 3 (species identity):

y~Block + Herbivory+ *G. pratense* (0,1) + *D. glomerata* (0,1) + (Herbivory* *G. pratense*) + (*D. glomerata* * Herbivory)

where y was either VOC emission of the *T. pratense* plant or of the entire plant community (either individual compounds or total compounds). Plant biomass and plant community herbivory (in this case without the variable herbivory as a predictor) were also tested with these models. Thus, for every analysis (using aov or glm), we always tested the three different models.

The Akaike information criterion (AIC) was used to determine the goodness of fit of the different models and to compare models [7]. Since each variable (y) was analyzed three times to test effects of species richness, composition and species identity, *p* values were adjusted using the false discovery rate (FDR) method to avoid false positives associated with multiple hypothesis testing.

There were three missing values in the community data and four missing values in the single plant measurements. This was due to low signal to noise ratios in the VOC measurements of these plants. Additionally, one value in the single plant measurements was considered an outlier and thus removed from the statistical analysis.

For ease of reading, results of the statistical analyses are summarized in tables, Note that in the main body of the text, tables are condensed to save space, more detailed versions of the statistical tables are given in the supplement.

## Additional files

Additional file 1: **Figure S1.** (A) Biomass of the focal plant *Trifolium pratense* growing in different mixtures and (B) biomass of entire experimental plant communities. The biomass of the focal *T. pratense* plants was affected by plant species richness, species composition and the identity of the neighbouring plants. *T. pratense* individuals growing in plant species mixtures had a lower biomass than individuals growing in monocultures. For entire plant communities, species richness, species composition and the identity of the neighbouring plants affected total biomass. Biomass was highest in the most diverse community and communities with *D. glomerata* were found to have higher biomass than other communities. **Table S1.** Statistical results for (A) biomass of the focal plant *Trifolium pratense* growing in different mixtures and (B) biomass of entire plant communities. **Figure S2.** Percent leaf area loss due to *Spodoptera littoralis* feeding on the focal *Trifolium pratense* plants growing in different mixtures. The percent leaf area loss due to *Spodoptera littoralis* caterpillar feeding was on average 18.4 ± 2.0%. Herbivory tended to increase with species richness, but this was not significant. Plant species composition and neighbor identity did not affect caterpillar herbivory. **Table S2.** Statistical results for herbivory on *Trifolium pratense* growing in different plant species mixtures. **Table S3.** Statistical results for the effect of herbivory on the emission of individual compounds from the headspace of (A) individual *Trifolium pratense* and (B) from the headspace of entire communities of different plant species mixtures. (DOCX 152 kb)

Additional file 2: **Table S4.** Full statistical results for the VOC emission of individual *Trifolium pratense* plants growing in different experimental plant communities (extension of Table 2). (DOCX 29 kb)

Additional file 3: **Table S5.** Full statistical results for the VOC emission of entire experimental plant communities (extension of Table 4). (DOCX 32 kb)

## Abbreviations

C: Non-infested control plants
DMNT: 4,8-dimethyl-1,3,7-nonatriene
H: Plants infested with *Spodoptora littoralis* caterpillars
HIPV: Herbivore induced plant volatiles
PET: Polyethylene terephthalate
T: *Trifolium pratense* monoculture
TD: Two-species mixture containing *T. pratense* and *Dactylis glomerata*
TDG: Three species mixture with *T. pratense*, *D. glomerata* and *G. pratense*
TG: Two-species mixture containing *T. pratense* and *Geranium pratense*
VOC: Volatile organic compounds
