# Supplementary material for: Plant volatile emission depends on the species composition of the neighboring plant community
Source: BMC Plant Biol. 2019 Feb 6;19:58. doi: 10.1186/s12870-018-1541-9 (PMC6366091; doi:10.1186/s12870-018-1541-9)
Supplement: Supplementary file 1 — Figure S1. (A) Biomass of the focal plant Trifolium pratense growing in different mixtures and (B) biomass of entire experimental plant communities. The biomass of the focal T. pratense plants was affected by plant species richness, species composition and the identity of the neighbouring plants. T. pratense individuals growing in plant species mixtures had a lower biomass than individuals growing in monocultures. For entire plant communities, species richness, species composition and the identity of the neighbouring plants affected total biomass. Biomass was highest in the most diverse community and communities with D. glomerata were found to have higher biomass than other communities. Table S1. Statistical results for (A) biomass of the focal plant Trifolium pratense growing in different mixtures and (B) biomass of entire plant communities. Figure S2. Percent leaf area loss due to Spodoptera littoralis feeding on the focal Trifolium pratense plants growing in different mixtures. The percent leaf area loss due to Spodoptera littoralis caterpillar feeding was on average 18.4 ± 2.0%. Herbivory tended to increase with species richness, but this was not significant. Plant species composition and neighbor identity did not affect caterpillar herbivory. Table S2. Statistical results for herbivory on Trifolium pratense growing in different plant species mixtures. Table S3. Statistical results for the effect of herbivory on the emission of individual compounds from the headspace of (A) individual Trifolium pratense and (B) from the headspace of entire communities of different plant species mixtures. (DOCX 152 kb) [file 12870_2018_1541_MOESM1_ESM.docx]

**Additional File 1**

Figures S1 and S2, Tables S1-S3

**

**

**Figure S1**: (**A**) Biomass of the focal plant *Trifolium pratense* growing in different mixtures. TG is the two-species-mixture with *T. pratense* and *Geranium pratense*. TD the two-species-mixture with *T. pratense* and *Dactylis glomerata*. Bars represent means ± SEM. n = 6. (**B**) Biomass of entire communities. For each community, the contribution of each species is shown as an average over all communities with a particular species richness (individual species biomass means ± SEM). White bars *T. pratense* (**T**), hatched bars *G. pratense* (**G**) and grey bars are *D. glomerata* (**D**). Note that only *T. pratense* was included as a monoculture. Bars represent means ± SEM, n = 6. Statistical results are presented in Table S1.

**Table S1:** Statistical results for (**A**) biomass of the focal plant *Trifolium pratense* growing in different mixtures (TG: two-species-mixture with *T. pratense* and *Geranium pratense*, TD: two-species-mixture with *T. pratense* and *Dactylis glomerata*) and (**B**) biomass of entire communities (Fig. S1). The results presented in the table show the effect of **species richness** (model 1, 1 to 3 plant species), **species composition** (model 2, four levels, *T. pratense* monoculture, species mixture of *T. pratense* and *Geranium pratense*, species mixture of *T. pratense* and *Dactylis glomerata* and species mixture containing *T. pratense*, *G. pratense* and *D. glomerata*), and **species identity** (model 3; presence of *D. glomerata* or presence of *G. pratense*). Interactions between the diversity treatment and the herbivory treatment are reported only when they were significant. Otherwise, they were excluded from the maximal model during model simplification *(Excl*.). Species richness, species composition and species identity (presence or absence of a species) were tested in separate analyses of variance models (models 1-3) following transformation of data to meet assumption of normality (see manuscript text for details, n/a: term not in model.). The Akaike information Criterion (AIC) is shown for all models. F-ratios given in bold are significant, asterisks indicate the level of significance: * p<0.05, ** p<0.01, *** p<0.001. Bold numbers in the last line (AIC) indicate the model with the lowest AIC among the three models (1-3).

|  |  |  |  |  |  |  |  |  |  |  |  |  |  |  |  |  |  |  |
| --- | --- | --- | --- | --- | --- | --- | --- | --- | --- | --- | --- | --- | --- | --- | --- | --- | --- | --- |
| **Variable** |  | **(A) Single *T. pratense* Biomass (g)** | | | | |  | **(B) Community Biomass (g)** | | | | |  | **(C) Herbivory (%)** | | | | |
|  |  | ***Model 1*** |  | ***Model 2*** |  | ***Model 3*** |  | ***Model 1*** |  | ***Model 2*** |  | ***Model 3*** |  | ***Model 1*** |  | ***Model 2*** |  | ***Model 3*** |
|  |  | ***F (1,36)*** |  | ***F (1,34)*** |  | ***F (1,35)*** |  | ***F (1,37)*** |  | ***F (1,35)*** |  | ***F (1,36)*** |  | ***F (1,36)*** |  | ***F (1,34)*** |  | ***F (1,35)*** |
| Herbivory (0,1) |  | n/a |  | n/a |  | n/a |  | n/a |  | n/a |  | n/a |  | n/a |  | n/a |  | n/a |
| Species richness |  | **5.44 *** |  | n/a |  | n/a |  | **6.54 *** |  | n/a |  | n/a |  | 1.98 |  | n/a |  | n/a |
| Species composition |  | n/a |  | **4.23 *** |  | n/a |  | **n/a** |  | **6.32** | ****** | n/a |  | n/a |  | 1.57 |  | n/a |
| *G. pratense* presence |  | n/a |  | n/a |  | *Excl.* |  | n/a |  | n/a |  | *Excl.* |  | n/a |  | n/a |  | 1.99 |
| *D. glomerata* presence |  | n/a |  | n/a |  | **6.71** |  | n/a |  | n/a |  | **17.72 ***** |  | n/a |  | n/a |  | *Excl.* |
| Herbivory*species richness |  | *Excl.* |  | n/a |  | n/a |  | *Excl.* |  | n/a |  | n/a |  | *Excl.* |  | n/a |  | n/a |
| Herbivory*species composition |  | n/a |  | *Excl.* |  | n/a |  | n/a |  | *Excl.* |  | n/a |  | n/a |  | *Excl.* |  | n/a |
| Herbivory**G. pratense* presence |  | n/a |  | n/a |  | *Excl.* |  | n/a |  | n/a |  | *Excl.* |  | n/a |  | n/a |  | *Excl.* |
| Herbivory**D. glomerata* presence |  | n/a |  | n/a |  | *Excl.* |  | n/a |  | n/a |  | *Excl.* |  | n/a |  | n/a |  | *Excl.* |
| AIC |  | -45.92 |  | **-49.5** |  | -45.91 |  | 128.68 |  | 120.8 |  | **120.23** |  | **-81.56** |  | -80.91 |  | -80.01 |
|  |  |  |  |  |  |  |  |  |  |  |  |  |  |  |  |  |  |  |

**Figure S2**: Percent leaf area loss due to *Spodoptera littoralis* feeding on the focal *Trifolium pratense* plants growing in different mixtures. TG is the two-species-mixture with *T. pratense* and *Geranium pratense*. TD the two-species-mixture with *T. pratense* and *Dactylis glomerata*. Plants were individually exposed to feeding by three *S. littoralis* caterpillars enclosed in a bag to prevent movement to other plants in the community. Bars represent means ± SEM, n = 6. Statistical results are presented in Table S2.

**Table S2:** Statistical results for **herbivory** on *Trifolium pratense* growing in different plant species mixtures (Fig. S2). The results presented in the table show the effect of **species richness** (model1, 1 to 3 plant species), **species composition** (model 2, four levels, *T. pratense* monoculture, species mixture of *T. pratense* and *Geranium pratense*, species mixture of *T. pratense* and *Dactylis glomerata* and species mixture containing *T. pratense*, *G. pratense* and *D. glomerata*), and **species identity** (model 3; presence of *D. glomerata* or presence of *G. pratense*). Interactions between the diversity treatment and the herbivory treatment are reported only when they were significant. Otherwise, they were excluded from the maximal model *(Excl*.). Species richness, species composition and species identity (presence or absence of a species) were tested in separate analysis of variance (ANOVA) models following transformation of data to meet assumption of normality (see manuscript text for details). The Akaike information Criterion (AIC) is shown for all models. F-ratios given in bold are significant, asterisks indicate level of significance: * p<0.05, ** p<0.01, *** p<0.001. The bold number in the last line (AIC) indicates the model with lowest AIC among the three models 1-3.

| **Variable** |  | **Herbivory (%)** | | | | |
| --- | --- | --- | --- | --- | --- | --- |
|  |  | ***Model 1*** |  | ***Model 2*** |  | ***Model 3*** |
|  |  | ***F (1,36)*** |  | ***F (1,34)*** |  | ***F (1,35)*** |
| Herbivory (0,1) |  | n/a |  | n/a |  | n/a |
| Species richness |  | 1.98 |  | n/a |  | n/a |
| Species composition |  | n/a |  | 1.57 |  | n/a |
| *G. pratense* presence |  | n/a |  | n/a |  | 1.99 |
| *D.glomerata* presence |  | n/a |  | n/a |  | *Excl.* |
| Herbivory*species richness |  | *Excl.* |  | n/a |  | n/a |
| Herbivory*species composition |  | n/a |  | *Excl.* |  | n/a |
| Herbivory**G. pratense* presence |  | n/a |  | n/a |  | *Excl.* |
| Herbivory**D. glomerata* presence |  | n/a |  | n/a |  | *Excl.* |
| AIC |  | **-81.56** |  | -80.91 |  | -80.01 |

**Table S3:** Statistical results for the **effect of herbivory on the emission of individual** **compounds** from the headspace of (**A**) individual *Trifolium pratense* and (**B)** from the headspace of entire communities of different plant species mixtures. The statistical results were isolated from the full models (models 1-3). F-ratios given in bold are significant, asterisks indicate level of significance: * p<0.05, ** p<0.01, *** p<0.001. Note that these values were extracted from full models (models 1-3). The full models are reported in other tables (Table 2 and Table 4) where appropriate, i.e. for many of the individual compounds we do not report the effect of species richness, composition or identity of neighbouring plants.

| **Single Compounds** |  | **Effect of herbivory on emission of VOCs** | | | |  |
| --- | --- | --- | --- | --- | --- | --- |
|  |  | ***(A) Individual Plants*** | |  | ***(B)***  ***Entire Community*** | |
|  |  | ***F(1,42)*** |  |  | ***F(1,42)*** |  |
| **Monoterpenes** |  |  |  |  |  |  |
| (*E*)-β-ocimene |  | **221.56** | ****** |  | 1.62 | ns |
| (*Z*)-β-ocimene |  | **49.77** | ****** |  | 1.29 | ns |
| **Sesquiterpenes** |  |  |  |  |  |  |
| (*E*)-β-caryophyllene |  | **15.98** | ** |  | **7.31** | ****** |
| **Homoterpenes** |  |  |  |  |  |  |
| DMNT |  | **51.93** | **** |  | **15.60** | ** |
| **GLVs** |  |  |  |  |  |  |
| (*Z*)-3-hexenyl acetate |  | 2.19 | ns |  | 0.9 | ns |
| **Others** |  |  |  |  |  |  |
| 1-Octene-3-ol |  | **5.52** | * |  | *3.83* | * |
| Benzyl alcohol |  | 3.05 | ns |  | 0.23 | ns |
| Unknown |  | 1.86 | ns |  | 0.96 | ns |
| Nonanal |  | 2.71 | ns |  | 0.00 | ns |
| **Total** |  | **39.91** | ****** |  | 0.12 | ns |
