# Supplementary material for: Plant volatile emission depends on the species composition of the neighboring plant community
Source: BMC Plant Biol. 2019 Feb 6;19:58. doi: 10.1186/s12870-018-1541-9 (PMC6366091; doi:10.1186/s12870-018-1541-9)
Supplement: Supplementary file 2 — Table S4. Full statistical results for the VOC emission of individual Trifolium pratense plants growing in different experimental plant communities (extension of Table 2). (DOCX 29 kb) [file 12870_2018_1541_MOESM2_ESM.docx]

**Additional File 2**

**Table S4:** Full statistical results for the VOC emission of individual *Trifolium pratense* plants growing in different experimental plant communities (extension of Table 2). This table is identical to table 2 except that here the full minimum adequate model is presented, i.e. all interactions. The results presented in the table show the effect of **species richness** (model1, 1 to 3 plant species), **species composition** (model 2 four levels, *Trifolium pratense* monoculture, species mixture of *T. pratense* and *Geranium pratense*, species mixture of *T. pratense* and *Dactylis glomerata* and species mixture containing *T. pratense*, *G. pratense* and *D. glomerata*) and **species identity** (model 3; *D. glomerata* presence or *G. pratense* presence). **A)** Results for **total VOC** emission of individual compound classes, in ng g^-1^ h^-1^ and **B)** results for the **relative amounts** of the major groups of volatiles presented with respect to the full odour blend of each community (%). Interactions between diversity treatments and herbivory treatments are reported only when they were significant. Otherwise, they were excluded from the maximal model *(Excl*.). Species richness, species composition and species identity (presence or absence of a species) were tested in separate analysis of variance (ANOVA) models following transformation of data to meet assumption of normality (see text for details). F-ratios given in bold are significant, stars (**) indicate level of significance: * p<0.05, ** p<0.01, *** p<0.001. Bold AIC indicates model with lowest AIC among models 1-3.

n/a not applicable (not tested)
