# Supplementary material for: Plant volatile emission depends on the species composition of the neighboring plant community
Source: BMC Plant Biol. 2019 Feb 6;19:58. doi: 10.1186/s12870-018-1541-9 (PMC6366091; doi:10.1186/s12870-018-1541-9)
Supplement: Supplementary file 3 — Table S5. Full statistical results for the VOC emission of entire experimental plant communities (extension of Table 4). (DOCX 32 kb) [file 12870_2018_1541_MOESM3_ESM.docx]

**Additional File 3**:

**Table S5:** Full statistical results for the VOC emission of entire experimental plant communities (extension of **Table 4**). This table is identical to table 4 except that here the full minimum adequate model is presented, i.e. all interactions. The results presented in the table show the effect of species richness (model1; 1 to 3 plant species), species composition (model 2; four levels, *Trifolium pratense* monoculture, species mixture of *T. pratense* and *Geranium pratense*, species mixture of *T. pratense* and *Dactylis glomerata* and species mixture containing *T. pratense*, *G. pratense* and *D. glomerata*) and species identity (model 3; *D. glomerata* presence or *G. pratense* presence) on the VOC emission (**Fig 1 C**) in (**A**) absolute amounts (ng g^-1^ h^-1^) and as (**B**) relative amounts of the major groups of volatiles presented with respect to the full odour blend of each community (%; **Fig 4**). Interactions between diversity treatments and herbivory treatments are reported only when they were significant. Otherwise, they were excluded from the maximal model *(Excl*.). Species richness, species composition and species identity (presence or absence of a species) were tested in separate analysis of variance (ANOVA) models following transformation of data to meet assumption of normality (see text for details). F-ratios given in bold are significant, stars (**) indicate level of significance: * p<0.05, ** p<0.01, *** p<0.001. Bold AIC indicates model with lowest AIC among models 1-3. Each comparison had at least 5 replicates.

n/a not applicable (not tested), *Excl.-* Excluded

n/a not applicable (not tested), *Excl.-* Excluded
